# Natural Products and Their Neuroprotective Effects in Degenerative Brain Diseases: A Comprehensive Review

**DOI:** 10.3390/ijms252011223

**Published:** 2024-10-18

**Authors:** Dong Wook Lim, Jung-Eun Lee, Changho Lee, Yun Tai Kim

**Affiliations:** Division of Functional Food Research, Korea Food Research Institute, Wanju 55365, Republic of Korea; dwlim@kfri.re.kr (D.W.L.); je_lee@kfri.re.kr (J.-E.L.); chang@kfri.re.kr (C.L.)

**Keywords:** degenerative brain disorders, Alzheimer’s disease, neuroprotection, natural products, medical plants

## Abstract

As the global population ages, the incidence of neurodegenerative diseases such as Alzheimer’s and Parkinson’s is rapidly rising. These diseases present a significant public health challenge, as they severely impair cognitive and motor functions, ultimately leading to a substantial reduction in quality of life and placing a heavy burden on healthcare systems worldwide. Although several therapeutic agents have been developed to manage the symptoms of these diseases, their effectiveness is often limited, and there remains an urgent need for preventive strategies. Growing evidence indicates that bioactive compounds from natural products possess neuroprotective properties through antioxidant and anti-inflammatory effects, modulating key pathways such as phosphatidylinositol 3-kinase/protein kinase B (PI3K/AKT) and brain-derived neurotrophic factor–tropomyosin receptor kinase B–cAMP response element-binding protein (BDNF-TrkB-CREB), which are crucial for neuronal survival. These compounds may also reduce amyloid-beta and tau pathology, as well as enhance cholinergic neurotransmission by inhibiting acetylcholinesterase activity. By targeting oxidative stress, neuroinflammation, and neurodegeneration, natural products offer a promising approach for both prevention and treatment. These findings suggest that natural products may be promising for preventing and treating neurodegenerative diseases. This review aims to explore the pathogenesis of neurodegenerative diseases, the limitations of current therapies, and the potential role of natural products as therapeutic agents.

## 1. Introduction

**Figure 1:**
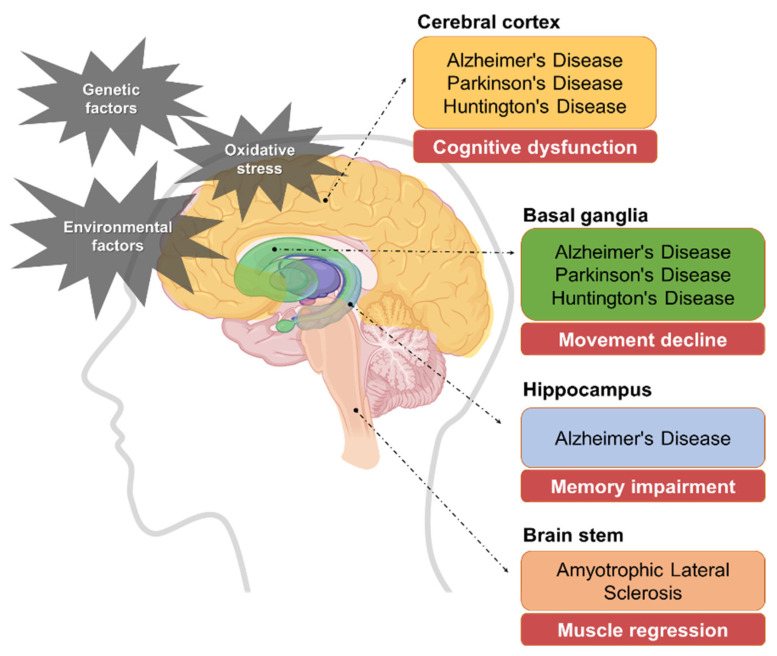
Degenerative brain diseases that occur mainly in each major brain region and their representative symptoms.

Recently, as the aging society accelerates, the incidence of degenerative brain diseases, commonly known as dementia, is rapidly increasing. The global social cost of degenerative brain diseases was estimated to be approximately USD 1313.4 billion for 55.2 million dementia patients in 2019, which corresponds to an annual cost of USD 23,796 per patient [1]. Additionally, according to a report by the World Health Organization (WHO), the current number of degenerative brain disease patients worldwide is estimated to be around 55 million. This number is expected to rise to approximately 80 million by 2030 and 140 million by 2050, clearly indicating that degenerative brain diseases are a very serious concern in our lives [2]. Degenerative brain diseases result from aging and involve a decline in brain cell function, reduction in neuronal cell number, neuronal cell death, and loss of synapse functionality [3]. While the exact causes remain unclear, factors such as neuronal cell dysfunctions contribute to the development of conditions like Alzheimer’s (AD), Parkinson’s (PD), Huntington’s disease (HD), and Amyotrophic Lateral Sclerosis (ALS) [4]. The main symptoms, pathological mechanisms, and treatment approaches of these diseases are summarized as follows in Figure 1 and Table 1.

Degenerative brain diseases, including AD, PD, HD, and ALS, have pathological mechanisms that collectively lead to neuronal damage and death. These mechanisms include oxidative stress and excessive activation of glutamate receptors, which contribute to neuronal injury [5]. The overproduction of ROS induces oxidative stress in neurons, generating neurotoxic substances like isoprostanes, which impair neuronal function and viability [6]. Indeed, mitochondrial dysfunction in neurons reduces adenosine triphosphate (ATP) production, essential for cell survival, and leads to cell death [7]. Overactivation of glutamate receptors increases intracellular calcium levels, causing excitotoxicity and neuronal damage [8]. Additionally, patients with these diseases show altered levels of antioxidant enzymes such as superoxide dismutase (SOD), catalase (CAT), and glutathione peroxidase (GPx) compared to healthy individuals, which changes with disease progression [9]. The intake of functional materials with antioxidant properties has shown potential in preventing or improving symptoms in patients with degenerative brain diseases [10]. It has been reported from randomized controlled trials that the consumption of Souvenaid^®^, as a medicinal food, improved memory performance after 12 weeks in patients with mild AD [11]. Administration of Lion’s mane mushroom (*Hericium erinaceus*) over 49 weeks resulted in significantly improved scores on the functional cognitive assessment scale (FUCAS), as well as enhanced contrast sensitivity, in patients with mild AD compared to the placebo group [12]. A 24-week, randomized, double-blind, placebo-controlled study using omega-3 polyunsaturated fatty acid (PUFA) monotherapy in mild AD patients showed better improvement on the FUCAS than those in the placebo group [13]. A daily dose of 500 mg of Melissa officinalis, also known as Lemon balm, containing rosmarinic acid for 24 weeks showed no significant differences in cognitive measures. However, the mean neuropsychiatric inventory questionnaire (NPI-Q) score improved in mild AD patients without any side effects [14]. The exact mechanism of action of these various natural products is not yet fully understood. However, various studies have reported that active compounds derived from natural products, recognized for their antioxidant or anti-inflammatory properties, inhibit neuronal cell death and significantly enhance cognitive function-related behaviors in animal models of neurodegenerative diseases [15]. Oxidative stress caused by excessive ROS production plays a crucial role in the development of neurodegenerative diseases. Factors like Aβ or tau protein accumulation, APOE ε4, and α-synuclein aggregates contribute to increased ROS, leading to mitochondrial dysfunction, cellular damage, and neuronal death. ROS disrupt neuronal functions by impairing BDNF expression, TrkB receptor activation, and the PI3K/AKT pathway, which are essential for neuronal survival and plasticity. Additionally, ROS activate inflammatory pathways, involving microglia and transcription factors like NF-κB, furthering oxidative stress and neuroinflammation. These processes create a vicious cycle of cellular damage and inflammation, suggesting that targeting ROS production or the inflammatory response could be vital for neuroprotection and treating neurodegenerative diseases. Consequently, these studies suggest that natural products may have potential therapeutic benefits for degenerative brain diseases. This review provides a concise overview of the pathogenesis and current treatments of degenerative brain diseases to enhance understanding in this field. It focuses on natural products with potential neuroprotective effects, detailing their mechanisms of action and evaluating their potential as alternative therapeutic strategies for degenerative brain conditions.

**Table 1:** The main symptoms, pathological mechanisms (including genetic factors), and treatment approaches of degenerative brain diseases.

Diseases	Main Symptoms	Pathological Mechanisms	Genetic Factors	Diagnostic Methods	Treatment Approaches	Life Expectancy (After Diagnosis Years)
Alzheimer’s Disease [16]	Memory loss, cognitive impairment, behavioral changes	Amyloid plaques and tau protein tangles	APOE ε4 allele	Clinical diagnosis, MRI, PET, cerebrospinal fluid tests	Symptomatic relief medications (cholinesterase inhibitors, NMDA receptor antagonists)	3–10 [17]
Parkinson’s Disease [18]	Tremors, rigidity, bradykinesia, balance problems	Loss of dopaminergic neurons, Lewy body formation	LRRK2, PARK7, PINK1, SNCA mutations	Clinical diagnosis, DAT scan	Dopamine replacement therapy, dopamine agonists, physical therapy	9– [19]
Huntington’s Disease [20]	Chorea (involuntary movements), cognitive decline, psychiatric symptoms	Abnormal accumulation of huntingtin protein	HTT gene mutation	Genetic testing	Symptomatic relief medications, behavioral therapy, physical therapy	15–20 [21]
Amyotrophic Lateral Sclerosis [22]	Muscle weakness, atrophy, respiratory difficulty	Loss of motor neurons	SOD1, C9orf72, TARDBP, FUS mutations	Clinical diagnosis, electromyography (EMG), nerve conduction studies	Symptomatic relief medications, respiratory support, physical therapy	3–5 [23]

APOE ε4 allele: apolipoprotein E epsilon 4 allele, NMDA: N-Methyl-D-Aspartate, LRRK2: Leucine-rich repeat kinase 2, PARK7: Parkinsonism-associated deglycase, PINK1: PTEN-induced putative kinase 1, SNCA: synuclein alpha (α-synuclein), HTT gene: huntingtin gene, SOD1: superoxide dismutase 1, C9orf72: Chromosome 9 open reading frame 72, TARDBP: TAR DNA-binding protein 43 (TDP-43), FUS: Fused in sarcoma.

## 2. Degenerative Brain Diseases

### 2.1. Alzheimer’s Disease

Alzheimer’s disease (AD) is characterized by the excessive deposition of amyloid-β (Aβ) and tau proteins in the brain [24]. Aβ is generated during the cleavage of amyloid precursor protein (APP) by β-site amyloid precursor protein cleaving enzyme 1 (BACE1) and γ-secretase [25]. Under normal conditions, Aβ is either removed or aids in the signaling and survival of neurons in the brain. However, the abnormal accumulation of Aβ leads to the formation of Aβ oligomers and subsequently Aβ plaques [26]. Aβ oligomers directly disrupt synaptic function, impairing synaptic plasticity, which is critical for cognitive processes [27]. Aβ plaques induce the production of reactive oxygen species (ROS) within neurons and cause excessive activation of microglia and astrocytes, triggering inflammatory responses that result in neuronal damage and death [28]. Additionally, Aβ plaques damage the blood–brain barrier (BBB), allowing impurities to penetrate the brain more easily, leading to neuroinflammation and causing direct damage to neurons [29]. It has been previously reported that the apolipoprotein E (APOE ε4) allele is a major factor in the development of AD, as this gene promotes the formation of Aβ plaques [30]. Tau proteins contribute to maintaining the morphology of neurons, particularly by stabilizing microtubules within axons and dendrites to support cellular structures [31]. When tau proteins in the brain become excessively phosphorylated, they detach from the microtubules and accumulate abnormally. These hyperphosphorylated tau proteins have very low solubility and form abnormal aggregates within neurons, which disrupt normal cellular functions and impair neural signaling, leading to neuronal damage and cell death [32]. For this reason, treatments for AD have focused on inhibiting the excessive deposition of Aβ and tau proteins, leading to the development of BACE1 inhibitors such as Verubecestat, Atabecestat, and Elenbecestat, which target β-secretase to cleave APP and inhibit the production of Aβ protein [33]. However, these BACE1 inhibitors have not demonstrated significant therapeutic efficacy in patients with advanced AD, and some previous studies have reported serious side effects such as cognitive worsening [34]. Recently, Aducanumab and Lecanemab, anti-amyloid therapies targeting Aβ plaques, were approved in the United States in 2021 and 2023, respectively [35]. Their mechanism of action involves directly or indirectly inhibiting the formation of Aβ plaques in the brain by targeting degraded Aβ in the bloodstream [36]. In addition, treatments for AD primarily focus on alleviating symptoms and slowing disease progression, with existing therapies mainly utilizing cholinesterase inhibitors and NMDA (N-Methyl-D-aspartate) receptor antagonists [37]. Acetylcholine is a neurotransmitter crucial for signal transmission between nerve cells and plays a significant role in cognitive function [38]. It has been reported that in patients with AD, acetylcholine levels are significantly reduced [39]. Representative cholinesterase inhibitors, such as Donepezil, Rivastigmine, and Galantamine, help maintain acetylcholine concentration in the synaptic gap, thereby improving cognitive function [40]. NMDA receptors are one of the glutamate receptors mainly found in the synapses of nerve cells, and they play an important role in learning and memory [41]. In AD, excessive activation of glutamate causes neurotoxicity, resulting in nerve cell damage and cognitive decline [42]. Therefore, NMDA receptor antagonists such as Memantine are used for moderate AD and prevent excessive excitation of nerve cells, thereby inhibiting nerve cell damage [43].

### 2.2. Parkinson’s Disease 

Parkinson’s disease (PD) is characterized by the abnormal accumulation of α-synuclein protein and dysfunction of dopaminergic neurons [44]. α-Synuclein is found in the synapses of neuronal cells, where it normally helps transmit signals between neurons in the brain. However, abnormal accumulation of α-synuclein leads to the formation of Lewy bodies, which interfere with cellular activity and result in the damage or death of neuronal cells [45]. Dopamine is a neurotransmitter essential for signal transmission and plays a crucial role in movement control. It has been reported that dopamine levels in patients with PD are significantly reduced compared to those in healthy individuals [46].

Treatments for PD have primarily been developed with a focus on maintaining dopamine levels or stimulating dopamine receptors [47]. Representative dopamine replacement therapies include Levodopa and Carbidopa, which are converted into dopamine in the brain, thereby improving motor functions [48]. Dopamine receptor agonists such as Pramipexole, Ropinirole, and Bromocriptine directly stimulate dopamine D2 receptors, mimicking the effects of dopamine [49]. Additionally, Monoamine Oxidase B (MAO-B) inhibitors, including Selegiline, help maintain dopamine levels by inhibiting its degradation [50]. In patients with PD, it has been reported that not only a significant decrease in dopamine levels but also excessive glutamate activation are major contributors to the disease’s pathology [51]. Therefore, Amantadine, an NMDA receptor antagonist, prevents damage to nerve cells and alleviates motor symptoms by suppressing excessive glutamate activation [52]. Recent research into therapeutics that inhibit or eliminate the accumulation of α-synuclein has been actively conducted, suggesting that these therapeutic approaches have the potential to treat the underlying cause of PD [53].

### 2.3. Huntington’s Disease

Huntington’s disease (HD) is caused by genetic factors, specifically through toxic mechanisms within neurons due to the abnormal expansion of CAG repeats in the huntingtin (HTT) protein [54]. This condition primarily results in the degeneration of neurons in the basal ganglia, a brain region responsible for major functions such as motor learning and control, cognitive functions, and emotional regulation [55]. A normal HTT gene typically exhibits about 10–35 CAG repeats; however, in individuals with HD, more than 36 repeats are observed [56]. One of the treatments for HD includes dopamine transporter inhibitors like Tetrabenazine, which help alleviate motor symptoms by depleting dopamine levels [57]. Additionally, antipsychotic medications such as Risperidone are employed to improve psychiatric and behavioral symptoms [58]. Notably, gene therapies such as RNA interference (RNAi) and antisense oligonucleotides (ASOs) show potential in suppressing the abnormal expression of the HTT protein, thereby potentially slowing or halting disease progression [59].

### 2.4. Amyotrophic Lateral Sclerosis

Amyotrophic Lateral Sclerosis (ALS), also known as Lou Gehrig’s disease, is primarily characterized by the degeneration and death of motor neurons, a key pathological feature of the disease. Motor neurons play a crucial role in controlling muscle movement, and their damage leads to muscle weakness and atrophy [60]. ALS affects both upper and lower motor neurons, resulting in symptoms such as muscle stiffness, muscle weakness, and muscle cramps [61]. The exact cause of ALS remains not fully understood, but genetic factors such as mutations in the superoxide dismutase 1 (SOD1) gene are well-documented. These mutations disrupt the function of an enzyme that neutralizes the harmful effects of reactive oxygen species (ROS) [62]. Environmental factors, including exposure to heavy metals, viral infections, and smoking, have also been implicated in the disease [63]. The pathophysiological mechanisms of ALS involve a complex interplay of genetic, molecular, and cellular factors. Key pathological processes include excitotoxicity due to excessive glutamate, oxidative stress, mitochondrial dysfunction, protein misfolding, and impaired axonal transport [64]. Additionally, neuroinflammation and glial cell dysfunction contribute to the progressive neuronal damage observed in ALS [65]. Representative treatments for ALS include Riluzole and Edaravone. Riluzole works by inhibiting the release of glutamate, thereby reducing neuronal damage [66], while Edaravone acts as an antioxidant, suppressing oxidative stress-induced neuronal damage [67]. Gene therapy, particularly aimed at correcting SOD1 gene mutations or suppressing the expression of this gene, offers potential to slow or halt the progression of ALS [68].

### 2.5. Limitations of Current Pharmacotherapies in Degenerative Brain Diseases

The current pharmacotherapies for neurodegenerative diseases face significant limitations. In AD, treatments such as cholinesterase inhibitors and NMDA receptor antagonists provide symptomatic relief but do not halt disease progression, with emerging disease-modifying agents showing limited efficacy and safety concerns [69]. PD management with dopaminergic agents, dopamine agonists, and MAO-B inhibitors effectively ameliorates motor symptoms initially, but long-term use leads to motor fluctuations, dyskinesias, and neuropsychiatric side effects without addressing the underlying dopaminergic neurodegeneration [70]. HD therapies like vesicular monoamine transporter inhibitors and antipsychotics mitigate chorea and psychiatric symptoms but often exacerbate other motor and cognitive impairments, with no agents currently capable of targeting the disease’s genetic and neurodegenerative pathology [71]. In ALS, glutamate modulators and antioxidants modestly extend survival and slow disease progression, yet they offer minimal symptomatic relief and do not significantly alter the course of motor neuron degeneration [72]. Across these conditions, the lack of neuroprotective or disease-modifying treatments underscores the urgent need for novel therapeutic strategies.

## 3. Oxidative Stress and Neuronal Damage in Neurodegenerative Diseases

Although there are various types of degenerative brain disease, oxidative stress caused by excessive ROS production, or by factors that promote ROS production in neuronal cells, is a key contributor to their development [73]. For instance, the accumulation of Aβ or tau proteins can lead to mitochondrial dysfunction, which in turn promotes ROS formation, ultimately resulting in cellular damage and death [74]. Similarly, the APOE ε4 that modulates the metabolism, aggregation, and deposition of the Aβ allele increases the vulnerability of cell membranes to oxidative stress, thereby further accelerating ROS production [75]. Additionally, α-synuclein aggregates have been shown to enhance intracellular ROS production, exacerbating dopaminergic neuronal damage [76]. ROS negatively affect the survival, growth, differentiation, and synaptic plasticity of neuronal cells, primarily by inhibiting the expression of brain-derived neurotrophic factor (BDNF) and disrupting the tyrosine phosphorylation of TrkB receptors. This interference suppresses receptor activation and, consequently, neuronal function [77]. Furthermore, ROS inhibit the phosphorylation of cAMP response element-binding protein (CREB), hindering its activation and subsequent nuclear transcriptional activity [78]. Increased ROS production and the subsequent inhibition of phosphoinositide-3 kinase/AKT (PI3K/Akt) signaling pathway activation have been reported as critical factors in the pathogenesis of neurodegenerative diseases [79]. The PI3K/Akt pathway is a critical regulator of neuronal survival, growth, differentiation, motility, and dendritic expansion, and it is particularly well-known for being activated by BDNF to enhance neuronal plasticity [80]. In contrast, the accumulation of Aβ associated with AD has been reported to impair the function of the PI3K/Akt pathway, leading to the loss of its inhibitory effect on glycogen synthase kinase 3β (GSK-3β) [81]. Consequently, the activation of GSK-3β increases, which in turn enhances pro-apoptotic signaling within neurons and reduces cell survival. Furthermore, the activation of GSK-3β promotes the hyperphosphorylation of tau proteins, which leads to the formation of neurofibrillary tangles, accelerating neuronal damage and death, thereby advancing the progression of AD [82]. Indeed, the aggregation of alpha-synuclein is strongly influenced by ionic strength, with studies demonstrating that elevated ionic strength accelerates its aggregation, a hallmark of neurodegenerative disorders [83]. Similarly, AD, the aggregation of S100 protein family members, including S100A8, S100A9, S100A12, and S100B, is modulated by divalent cations such as Ca^2+^, which play a pivotal role in neuroinflammatory processes [84]. Additionally, metal ions (Cu^2+^, Fe^3+^, Zn^2+^, and Ca^2+^) are implicated in promoting tau oligomerization, contributing to the accumulation of ROS and exacerbating neurotoxicity [85]. Furthermore, ROS can alter the redox state and concentration of these ions, modifying ionic strength and thereby facilitating protein aggregation, which accelerates the formation of neurotoxic fibrils. These mechanisms are considered critical in the pathogenesis of neurodegenerative diseases.

**Figure 2:**
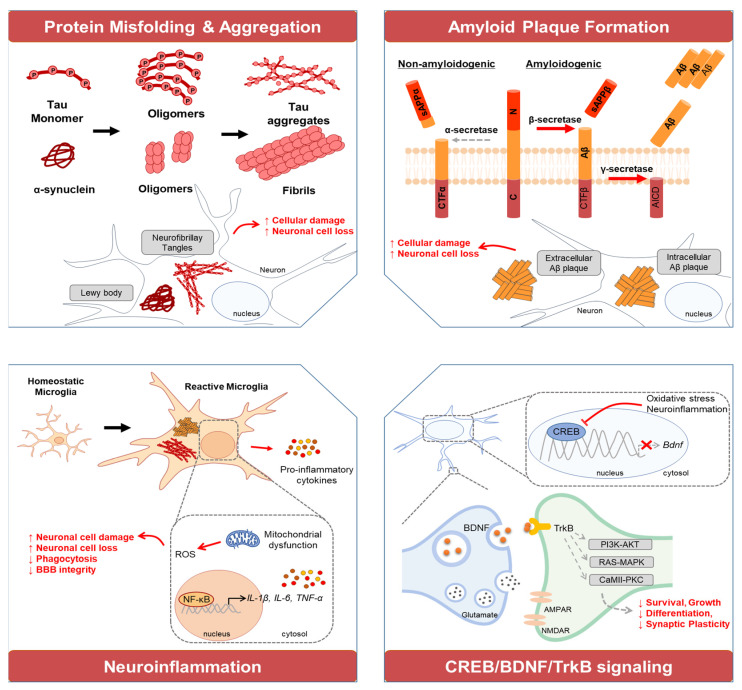
Oxidative stress and its role in the pathogenesis of degenerative brain diseases. Degenerative brain diseases are largely driven by oxidative stress caused by excessive ROS production, often triggered by factors like mitochondrial dysfunction, protein accumulation (Aβ, tau, α-synuclein), and genetic risk factors like APOE ε4. ROS impair neuronal survival, differentiation, and synaptic plasticity by disrupting key signaling pathways such as BDNF/TrkB and PI3K/AKT, leading to neuronal damage and cell death. They also activate microglia and inflammatory responses, creating a cycle of oxidative stress and inflammation. Additionally, ROS impair mitochondrial function and BBB integrity, further exacerbating neuronal damage and disease progression. sAPPα: soluble amyloid precursor protein alpha, sAPPβ: soluble amyloid precursor protein beta, N: N-terminus, CTFα: C-terminal fragment alpha, C: C-terminus, CTFβ: C-terminal fragment beta, AICD: amyloid precursor protein intracellular domain, Aβ: amyloid beta, ROS: reactive oxygen species, NF-κB: nuclear factor kappa B, IL-1β: Interleukin-1 beta, IL-6: Interleukin-6, TNF-α: Tumor necrosis factor alpha, BDNF: brain-derived neurotrophic factor, TrkB: tropomyosin receptor kinase B, AMPAR: α-amino-3-hydroxy-5-methyl-4-isoxazolepropionic acid receptor, NMDAR: N-methyl-D-aspartate Receptor, PI3K: phosphoinositide 3-Kinase, AKT: protein kinase B, RAS: Rat Sarcoma virus, MAPK: mitogen-activated protein kinase, CaMII: calcium/calmodulin-dependent protein kinase II, PKC: protein kinase C.

Additionally, ROS activate microglia in the brain, which in turn secrete pro-inflammatory cytokines, further amplifying oxidative stress. Moreover, ROS activate transcription factors such as NF-κB, promoting the expression of inflammatory genes that exacerbate the inflammatory response and induce additional ROS production [86]. The NF-κB cascade is activated to produce beta-Secretase 1 (BACE1)—an essential enzyme in producing Aβ oligomers [87]. This creates a vicious cycle of inflammation and oxidative damage. ROS also impair mitochondrial function, leading to decreased ATP production and further ROS generation. Mitochondrial dysfunction not only exacerbates the inflammatory response but also increases the production of inflammatory mediators [88]. Furthermore, ROS compromise the integrity of the blood–brain barrier, allowing external inflammatory mediators and immune cells to infiltrate the brain. This influx amplifies the brain’s inflammatory response and intensifies neuronal damage [89]. In conclusion, the interplay between ROS production and neuroinflammatory responses constitutes a major pathological mechanism that accelerates neuronal damage. Therefore, strategies aimed at inhibiting ROS production or modulating the inflammatory response may play a crucial role in neuroprotection and the treatment of neurodegenerative diseases (Figure 2).

## 4. Neuroprotective Activity of Medicinal Plants 

**Figure 3:**
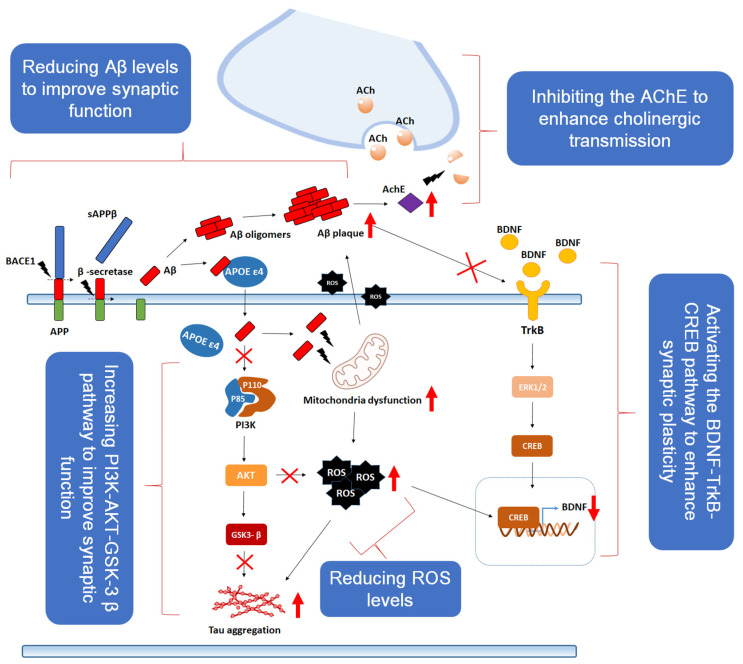
A natural product with well-established neuroprotective potential can exert its effects through multiple mechanisms. These mechanisms include AChE inhibition to enhance cholinergic transmission, a reduction in Aβ peptide and tau protein levels, and activation of the PI3K-AKT-GSK-3β pathway to improve synaptic function. Additionally, antioxidant and anti-inflammatory properties contribute to neuronal protection, while activation of the BDNF-TrkB-CREB pathway promotes synaptic plasticity. Furthermore, natural products may ameliorate synaptic dysfunction caused by oxidative stress and ROS generation. BACE1: beta-site amyloid precursor protein cleaving enzyme 1, APP: amyloid precursor protein, sAPPβ: soluble amyloid precursor protein beta, APOE ε4: apolipoprotein E epsilon 4 allele, Ach: acetylcholine, AchE: acetylcholinesterase, P85: phosphoinositide 3-kinase regulatory subunit p85, P110: phosphoinositide 3-kinase catalytic subunit p110, PI3K: hosphoinositide 3-kinase, AKT: protein kinase B, GSK3-β: glycogen synthase kinase 3 beta, ROS: reactive oxygen species, BDNF: brain-derived neurotrophic factor, TrkB: tropomyosin receptor kinase B, ERK1/2: extracellular signal-regulated kinase 1/2, CREB: cAMP response element-binding protein.

Based on the previously described neuroprotective mechanisms, one or more pathways may provide potential neuroprotective effects in preclinical studies. These pathways include inhibiting the enzyme acetylcholinesterase (AChE) to enhance cholinergic transmission, reducing Aβ peptide and tau protein levels, and activation of the PI3K-AKT-GSK-3β pathway to improve synaptic function, as well as antioxidant and anti-inflammatory activities that protect neurons. Additionally, activating the BDNF-TrkB-CREB pathway to enhance synaptic plasticity may also contribute to neuroprotection. These effects are present in natural substances containing various individual compounds, suggesting that natural products, including medicinal plants, may possess potential neuroprotective efficacy. Here, we summarize the potential neuroprotective effects and related mechanisms of various natural products, providing information to enhance the understanding of their potential use in the prevention or treatment of neurodegenerative diseases (Figure 3). In addition, we provide a brief overview of natural products that have demonstrated relevant mechanisms in recent studies, summarizing them in the final section (Table 2).

### 4.1. Allium cepa

*A. ceta* (onion) is one of the most important condiment plants and is consumed all over the world, and it is known for its various therapeutic and pharmacological effects, including antimicrobial, antioxidant, anti-inflammatory, and neuroprotective effects. It belongs to the Amaryllidaceae family and is rich in folic acid, vitamin B6, magnesium, calcium, potassium, and minerals [90]. In relation to neuroinflammation, a key pathogenesis of neurodegenerative disease, an in vitro study demonstrated that in the BV2 microglial cell line, *A. cepa* extract dose-dependently attenuated LPS-induced NO production and cytotoxicity, as well as the upregulation of iNOS, COX-2, pro-inflammatory cytokines, TNF-α, IL-1β, and IL-6 expression. Additionally, A. cepa extract provided protection against 1-methyl-4-phenylpyridinium (MPP+)-induced cell death in N27-A cells by mediating antiapoptotic gene Bcl-2 and enhancing antioxidant enzymes such as HO-1, NQO1, and catalase [91]. Research results from an AD-like model show that the ethyl acetate fraction of *A. cepa* exhibited neuroprotective effects against Aβ-induced ROS accumulation and cytotoxicity in PC12 cells. Additionally, its ameliorative effects have been shown to improve learning and memory behaviors in Aβ-induced cognitive dysfunction mouse models by protecting mitochondrial function and improving the cholinergic system [92]. In another AD-like model, a streptozotocin-induced rodent model also exhibited significant learning and memory impairments, along with oxidative stress and a cholinergic deficit in brain, while treatment with *A. cepa* reversed these deficits through acetylcholinesterase inhibition and antioxidant activity [93]. The cerebral ischemia–reperfusion injury model in mice, which mimics the clinical manifestations of human stroke, results in memory and sensorimotor dysfunctions, oxidative stress in the brain, and cerebral infarcts. However, treatment with *A. cepa* outer scale extract reversed these deficits through its antioxidant properties [94].

### 4.2. Arctium lappa

The root of *A. lappa*, commonly known as burdock, has long been cultivated in Asia as a folk remedy and edible plant. Several studies have reported that *A. lappa* has various pharmacological activities including antioxidant, anti-inflammatory, and neuroprotective activities. Treatment with *A. lappa* extract improved performance in Y-maze and passive avoidance tests in mice with Aβ-induced memory deficits [95]. Arctigenin from *A. lappa* extract prevented LPS-induced neuronal/synaptic injury and inhibited the increases in Aβ generation and the levels of APP and BACE1 [96]. Also, arctigenin has been reported to inhibit Aβ oligomers by suppressing the AKT/mTOR signaling pathway in APP/PS1 transgenic AD model mice [97].

### 4.3. Panax ginseng

*P. ginseng*, which is well-known as a traditional herbal medicine, has been reported in numerous preclinical and clinical studies to improve cognitive function and alleviate AD symptoms. Administration of fermented ginseng extract in doses of 400 or 800 mg/kg in a mouse model of AD significantly improved memory function-related behaviors using the passive avoidance test and Morris water maze, and it markedly reduced levels of Aβ42 protein in the brain of AD transgenic mice [98]. Indeed, Aβ oligomer-induced neuronal damage in mice treated with ginseng extract at doses of 100 and 500 mg/kg showed restoration of reduced synaptophysin and AChE intensity in the hippocampus, indicating that ginseng extract reverses memory impairment in AD [99]. Ginsenosides, also called ginseng saponins, exhibited neuroprotective activities such as anti-inflammation, antioxidation, antiapoptosis, hormone-like effects, and gut microbiota regulation [100]. Recently, a 12-week ginseng supplementation demonstrated improved memory in subjects aged 55 to 75 years with subjective memory impairment, with significant differences in cognitive improvement and AChE levels observed in a randomized, double-blind, placebo-controlled clinical trial [101].

### 4.4. Stephania japonica

The plant *S. japonica* is a common scrambler in the Menispermaceae family, primarily found in Bangladesh, where it has been traditionally used to treat vertigo, headaches, and sleep disorders. Treatment with *S. japonica* extract restored learning and memory in scopolamine-induced memory-impaired mice by significantly reducing AChE activity and oxidative stress [102]. The total alkaloid fraction from *S. japonica* demonstrated anti-neuroinflammatory activity via inhibition of microglia activation in middle cerebral artery occlusion (MCAO)-induced brain injury in rats [103]. Stepharine, a proaporphine alkaloid from *S. japonica,* also showed neuroprotective effects in a rat model of MCAO and lipopolysaccharide (LPS)-stimulated BV-2 cells [104]. Moreover, *S. japonica* exhibited neuroprotective effects against rotenone-induced neurotoxicity in SH-SY5Y cells [105].

### 4.5. Cucuma longa

*C. longa*, commonly known as turmeric, belongs to the Zingiberaceae family, which is known for its aromatic rhizomes and includes other notable plants like ginger and cardamom, and it has been widely recognized as a traditional herbal remedy with numerous health benefits [106]. *C. longa* extract, in doses of 100 or 200 mg/kg, has shown neuroprotective effects on scopolamine-induced memory impairment mice, evidenced by improved behaviors in the passive avoidance and Moris water maze tests by reducing AChE activity and inducing BDNF-CREB expression [107]. Administration of 200 mg/kg of *C. longa* extract exhibited neuroprotective effects on trimethyltin (TMT)-exposed SD rats by preventing oxidative stress [108]. Curcumin, a major active component from *C. longa*, has been identified as having various antioxidant and anti-inflammatory effects, and it has also been reported to inhibit AChE activity while exhibiting neuroprotective effects [109]. Indeed, a mixture including cucumin significantly inhibited the neuroinflammatory stimulus induced by Aβ exposure in THP-1 cells [110]. It has also been reported that in a randomized, placebo-controlled, double-blind study, the administration of a curcumin formulation to adults aged 40–90 years for 12 months resulted in significant changes observed through the Montreal Cognitive Assessment [111].

### 4.6. Withania somnifera

*W. somnifera* (Ashwagandha), a medicinal plant used in Indian traditional medicine for over 3000 years, is classified as a Rasayana in Ayurveda, where it is believed to enhance disease resistance, delay aging, and promote mental well-being. Clinical and preclinical studies support its potential in the treatment of anxiety and cognitive and neurological disorders, including Alzheimer’s and Parkinson’s diseases [112]. *W. somnifera* has been shown to improve memory in Bisphenol A (BPA)-induced cognitive dysfunction in mice by significantly increasing N-methyl-D-aspartate (NMDA) receptor levels through the inhibition of lipid peroxidation in the hippocampus [113]. Withanone, a compound derived from *W. somnifera*, improved cognitive impairment by inhibiting Aβ-42 accumulation and reducing elevated levels of pro-inflammatory cytokines such as TNF-α, IL-1β, IL-6, MCP-1, nitric oxide, and lipid peroxidation, as well as β- and γ-secretase enzymatic activity [114]. Indeed, *W. somnifera* exhibits neuroprotective effects in an MCAO model of ischemic stroke in mice [115].

### 4.7. Moringa oleifera

*M. oleifera*, known as the drumstick or horseradish tree, belongs to the family Moringaceae and has multiple bioactivities, including neuroprotective, anti-inflammatory, antidepressant, and cytotoxic effects [116]. In neuronal cells, *M. oleifera* extract showed neuroprotective effects through its antioxidative properties [117]. Moreover, *M. oleifera* extract has been shown to improve learning and memory deficits in rodent models of memory impairment [118]. It also prevented the loss of synaptic proteins and dendritic spines, facilitated the clearance of Aβ in the hippocampus, and mitigated cognitive dysfunction in APP/PS1 transgenic mice [119]. Furthermore, *M. oleifera* leaves extracts have exhibited antidepressant and anxiolytic effects, which are mediated through neurotransmitter pathways [120]. Additionally, isolated compounds from *M. oleifera*, such as moringin, astragalin, and isoquercitrin, along with identified compounds including phenolic acids and flavonoids (chlorogenic acid, gallic acid, ferullic acid, caffeic acid, kaempferol, quercetin, myrecetin, (-)-epicatechin, and isoquercitrin), have demonstrated significant neuropharmacological activities such as anti-neuroinflammatory and neuroprotective effects [121].

### 4.8. Melissa officinalis

*M. officinalis* L., commonly known as lemon balm, honey balm, or balm mint, is an edible medical herb belonging to the mint family Lamiaceae and the superfamily Nepetoideae [122]. *M. officinalis* has been shown to exhibit neuroprotective effects against apoptosis and mitochondrial dysfunction induced by neurotoxicity [123,124]. Additionally, its antioxidant properties have demonstrated protective effects in both in vivo and in vitro models of ischemic injury [125]. Previous studies have explored the impact of *M. officinalis* on cognitive function. In rodent models of memory impairment, induced either by Aβ via i.c.v. injection or by scopolamine administration, treatment with *M. officinalis* significantly improved memory function [126]. In another cognitive impairment model, the streptozotocin-induced rodent model, treatment with *M. officinalis* reversed the learning and memory deficits observed in the Y-maze and passive avoidance task, accompanied by increased expression of BDNF and NOS in the hippocampus [127]. An extract of *M. officinalis* also exhibits anxiolytic and anti-depressant effects, potentially mediated by its antioxidant and anti-apoptotic properties [128]. Indeed, clinical studies showed that supplementation of *M. officinalis* alleviated stress, anxiety, depression, and sleep disorder in burn patients and chronic stable angina [129].

### 4.9. Salvia officinalis

*S. officinalis* is a plant in the Lamiaceae family and has been reported to possess pharmacological properties, including anti-inflammatory, antinociceptive, antioxidant, cognitive-enhancing, and protective effects against neurodegenerative diseases [130]. Studies have shown that *S. officinalis* extract ameliorates learning and memory deficits in rodent models of cognitive impairment induced by diabetes or scopolamine, with concurrent antioxidant effects [131]. Furthermore, both the extract of S. officinalis and its components have demonstrated cholinesterase-inhibiting properties both in vitro and in vivo [132,133]. Consistent with these findings, supplementation with *S. officinalis* has been reported to enhance memory and attention in patients with mild to moderate Alzheimer’s disease [134]. Additionally, *S. officinalis* extract has shown potential therapeutic effects in rodent models of psychiatric disorders such as depression and anxiety [135].

### 4.10. Rhodiola crenulata

Rhodiola species, belonging to the Crassulaceae family and widely recognized in traditional medicine across Asian and Eastern European countries, exhibit beneficial properties, including, antidepressant, neuroprotective, antihypoxic, and antifatigue activities [136]. Notably, *R. crenulata* has demonstrated significant antioxidant activity and preventive effects against hypoxic damage [137]. Hypoxic damage causes impaired cognitive functions in healthy adults, while treatment with *R. crenulata* has proved beneficial for cognitive function under hypoxic conditions [138]. Additionally, the administration of *R. crenulata* attenuated hypoxia-induced brain injury in rodent models by reducing ROS accumulation and the number of apoptotic cells [139,140]. Further supporting its neuroprotective effects, *R. crenulata* extract significantly increased acetylcholine levels and choline acetyltransferase activity and decreased ROS accumulation in the brain of Aβ1-42 injected rodent models, thereby alleviating learning and memory deficits in these AD model animals [141]. Moreover, in D-galactose-induced aging rodent models, neuronal apoptosis was observed in the cortex and hippocampus; however, R. crenulata exhibited anti-apoptotic effects by increasing the phosphorylation of phosphoinositide-3 kinase/AKT (PI3K/AKT) in neurons [142].

### 4.11. Cinnamomum verum

Cinnamon and its essential oil are predominantly used as a spice and a food additive, yet they are also valued for their potent medicinal properties and applications in healthcare. They exhibit various pharmacological activities, including antioxidant, antibacterial, and immunomodulatory effects, as well as cognitive function enhancement, and they demonstrate potential therapeutic benefits for neurodegenerative disease such as AD [143]. For example, the essential oil from *C. verum* demonstrated potent activity against AChE and butyrylcholinesterase (BChE), suggesting its potential for managing patients of AD [144]. The oral administration of aqueous cinnamon extract demonstrated neuroprotective effects in an aluminum chloride (AlCl_3_)-induced AD rat model by inhibiting the formation of Aβ plaques in the cerebellum [145]. Eugenol, an active compound in cinnamon, significantly reversed memory deficits in the AlCl_3_-induced AD rat model [146]. Furthermore, in an in vitro model of PD, pretreatment with *C. verum* and its main bioactive component, cinnamaldehyde, effectively blocked 6-hydroxydopamine (OHDA)-induced apoptosis and reactive oxygen species (ROS) accumulation in PC12 cells [147].

### 4.12. Vitis vinifera

*V. vinifera* (grape) is one of the most widely cultivated fruit in the world, consumed fresh, dried, or processed into wine, and is valued in traditional medicine for its high content of polyphenols, vitamins, minerals, and organic acids. Notably, most grape polyphenols, including resveratrol, can cross the BBB in sufficient concentrations to evoke neuroprotective effects and cognitive enhancement by inhibiting acetylcholinesterase activity and facilitating the clearance of Aβ deposits [148]. Additionally, *V. vinifera* extract exhibits protective effects against glutamate-mediated oxidative stress-induced cell death in HT22 cells by upregulating antioxidant enzymes, including catalase, superoxide dismutases (SODs), glutathione S-transferases (GSTs), and glutathione peroxidase (GPx) [149]. Vitisin A, a resveratrol tetramer derived from the stembark of *V. vinifera*, exhibited significant neuroprotective effects by mitigating H_2_O_2_-induced cytotoxicity in SH-SY5Y cells and alleviating scopolamine-induced cognitive deficits in rodent models in behavioral assessments, through the upregulation of BDNF-CREB signaling and long-term potentiation [150].

### 4.13. Magnolia officinalis

Neolignans, including magnolol and honokiol, the main active constituents of *M. officinalis* cortex, have demonstrated neuroprotective effects through regulation of neuronal function and suppression of neurotoxicity, highlighting their potential for the prevention and treatment of neurological diseases [151]. It was reported that oral pretreatment with *M. officinalis* extract for 3 months inhibited memory impairment and Aβ accumulation in the brains of Tg2576 mice, an AD model [152]. Notably, honokiol is capable of crossing the BBB to exert therapeutic effects on CNS-related diseases [153]. Indeed, Honokiol significantly ameliorated cognitive impairment, synaptic damages, oxidative stress, and mitochondrial dysfunction in the hippocampus of APP/PS1 AD model mice [154]. Also, honokiol significantly facilitated the differentiation of primary oligodendrocyte precursor cells into mature oligodendrocytes by increasing the Akt/mTOR pathway [155].

### 4.14. Myristica fragrans

*M. fragrans* belongs to the Myristicaceae family and is extensively used in Indian traditional medicines to treat various diseases [156]. Research has shown that M. fragrans showed neuroprotective effects in H_2_O_2_-induced cell death in PC12 neuronal cells and inhibitory effects of butylcholinesterase (BChE) [157]. The methanolic extract of *M. fragrans* seeds has further demonstrated neuroprotective effects against scopolamine-induced oxidative damage, inflammation, and apoptosis in rat cortical tissue by reversing toxicity markers and enhancing antioxidant activity [158]. Compounds in *M. fragrans* exhibit anti-cholinesterase activity and modulate neurotransmitter levels [159]. The main components, myristicin, myristic acid, and elemicin, have demonstrated protective effects in animal models of epilepsy by attenuating neuronal loss and glial activation [160]. Additionally, treatment with *M. fragrans* seeds extract also alleviated motor behavioral impairments in rotenone-induced PD, potentially due to its anti-inflammatory, antioxidant, and anti-epileptic properties in the dopaminergic neurons [161].

### 4.15. Vaccinium angustifolium

Several species of berries are rich in polyphenols, particularly flavonoids, which are found in both are fruits and leaves. These compounds exhibit antioxidant and anti-inflammatory properties, offering potential benefits in mitigating brain aging and addressing neurodegenerative diseases [162]. *V. angustifolium*, commonly known as the wild blueberry, is recognized as one of the richest sources of phenolic compounds and noted for its high antioxidant content. Extracts from both the fruit and leaves of *V. angustifolium* have been shown to inhibit glutamate or α-synuclein-induced cell death and reduce morphological changes associated with inflammation [163]. Additionally, administration of extracts from *V. angustifolium* in rat models of cerebral ischemia/reperfusion injury demonstrated a reduction in oxidative stress and nitric oxide levels in both the hippocampus and serum, as well as an enlargement of the CA1 and CA3 regions of the hippocampus. These effects were accompanied by downregulation of iNOS/TNF-α and upregulation of miR-146a and miR-21 expression in the hippocampus [164]. Another study demonstrated that polyphenol extracts from *V. angustifolium* and *Vitis vinifera* (French grape) exhibit anti-Alzheimer’s effects. These extracts showed protective effects against AlCl_3_-induced increases in phosphorylated tau levels and Aβ accumulation in the hippocampus, resulting in improvements in learning and memory behaviors [165].

### 4.16. Tinospora cordifolia

*T. cordifolia*, which belongs to the Menispermaceae family, possess a range of medicinal properties, including antioxidant, anti-inflammatory, anti-stress, and immunomodulatory effects. The phytochemical components identified in *T. cordifolia*, including various polysaccharides, have been shown to exhibit immunomodulatory activity in many studies [166]. Extracts and compounds from *T. cordifolia* have the potential to aid in the management of neurodegenerative disease by restoring the antioxidant defense system and scavenging the free radicals [167]. Indeed, the butanol extract of *T. cordifolia* demonstrated protective effects on hippocampal neurons, both in vitro and in vivo, against glutamate-induced cell death and impaired plasticity through the upregulation of the ERK and AKT signaling pathway [168]. Furthermore, pretreatment with *T. cordifolia* in a rotenone-induced mouse model of PD ameliorated dopaminergic neuronal death and motor dysfunction, involving changes in gene expression related to neuroinflammation (e.g., NF-κB and TNF-α), mitochondrial dysfunction (e.g., Tfam), and neurotoxicity (e.g., Grin2A and Tiam) [169]. *T. cordifolia* also demonstrated improvements in memory status, reduced oxidative stress, and AChE inhibition in a rat model of AlCl_3_-induced neurotoxicity [170].

### 4.17. Arbutus unedo

*A. unedo*, commonly known as the strawberry tree, belongs to the Ericaceae family and is widely distributed in the Mediterranean region. Extracts of *A. unedo* and its components have various pharmacological properties including anti-inflammatory, antioxidant, antimicrobial, and neuroprotective activities [171]. The fruits of *A. unedo* are rich in polyphenolic compounds, such as phenolic acids, flavonoids, anthocyanins, coumarins, and quinones, providing them with high antioxidant potential, which may contribute to the prevention and management of neurodegenerative disease [172]. The essential oils from the leaves of *A. unedo* exhibit antioxidant activity and anti-inflammatory activity, as well as anti-acetylcholinesterase activity, which is associated with potential applications in AD treatment [173]. Arbutin, one of the bioactive compounds found in *A. unedo*, has been shown to reduce oxidative stress accumulation in the brain and alleviate cognitive dysfunction in a rat neurotoxicity model induced by monosodium L-glutamate (MSG) [174]. Furthermore, ellagic acid, a dietary polyphenol present in strawberry fruits, has been widely investigated in vivo, with numerous studies reporting its neuroprotective effects against a range of neurotoxins in animal models [175].

### 4.18. Juglans regia

*J. regia* belongs to the Juglandaceae family and is extensively used in traditional medicine for a variety of diseases, including neurodegenerative diseases. Treatment with *J. regia* extract in an Aβ-induced cognitive impairment model showed a protective effect on the antioxidant system and cholinergic system, resulting in the amelioration of learning and memory deficits. These effects were accompanied by changes in the expression levels of TNF-α, TNFR1, p-JNK, p-IκB, COX-2, IL-1β, p-Akt, and caspase-3 expression in cerebral tissues [176]. Walnut refers to the seed of the *J. regia*. Many reports have demonstrated that active peptides found in walnut have anti-Alzheimer’s disease effects by inhibiting Aβ fibrillation and oxidative stress [177]. Indeed, walnut protein hydrolysate (WPH) and its low-molecular-weight fraction (WPHL) ameliorate LPS-induced oxidative stress and memory deficits in mouse models. Furthermore, purified peptides from WPHL significantly reduce pro-inflammatory cytokines, including IL-6, IL-1β, and TNF-α, as well as ROS production and mitochondrial dysfunction in LPS-treated BV2 cells [178]. D-galactose-induced cognitive impairment mice exhibited memory deficits, while co-treatment with walnut-derived peptides reversed these impairments by ameliorating blood–brain barrier disruption [179].

### 4.19. Acanthopanax senticosus

*A. senticosus* is a widely used traditional Chinese herb, and its active compounds exhibit various biological activities, including neuroprotective, antioxidant, and anti-fatigue effects [180]. *A. senticosus*, previously classified as *Eleutherococcus senticosus*, is similar to ginseng, as both belong to the Araliaceae family. *A. senticosus* has positive pharmacological effects on the central nervous and immune systems and is associated with the PI3K/AKT signaling pathway [181]. Recent reports have demonstrated the preventive effects of *A. senticosus* extract against neurodegenerative disease extracts. Administration of *A. senticosus* extract in the MPTP-induced PD model significantly improved motor coordinate and altered protein expression in the PI3K/AKT signaling pathway and insulin receptor signaling pathway in cerebral tissues [182]. Furthermore, treatment with *A. senticosus* in the 5xFAD transgenic AD model significantly ameliorated learning and memory deficits, accompanied by the reversal of mRNA expression of APP, NTRK1, EGFR, ESR2, GSK3B, PAK1, and the MAPK signaling pathway in the hippocampus [183]. Treatment with capsules containing *A. senticosus*, Hirudo, Astmgali Radix, and other components in an APP/PS1 transgenic Alzheimer’s mouse model reduced Aβ plaque accumulation, p-Tau expression, cell death, and neuroinflammation in the brain [184].

### 4.20. Rosmarinus officinalis

*R. officinalis*, a well-known aromatic herb, has been used extensively in traditional medicine. Numerous studies have demonstrated that *R. officinalis* and its secondary metabolites have neuroprotective properties through various molecular mechanisms, including the inhibition of cholinesterase, modulation of the dopaminergic system, and regulation of oxidative and inflammatory pathways [185]. *R. officinalis* is also known for its potential in the prevention and treatment of neurodegenerative disease such as AD and PD by attenuating neuronal apoptosis, promoting neurogenesis, and enhancing cognitive function [186]. In a rat model of scopolamine-induced AD-like conditions, the administration of *R. officinalis* oil significantly improved working memory and long-term memory, accompanied by increased hippocampal neurogenesis and BDNF levels in serum and the hippocampus [187]. Rosmainic acid, a polyphenolic compound isolated from *R. officinalis*, exhibits a wide spectrum of biological activities. In a mouse model of MPTP-induced PD, treatment with rosmarinic acid increased dopaminergic signaling and prevented hyperlocomotion [188]. Moreover, *R. officinalis* is also well-known for its memory-enhancing effects, demonstrated both in vitro and in vivo, through the reduction in oxidative stress and the enhancement of the antioxidant system by modulating pathways such as Keap1/Nr2 [189].

**Table 2:** Natural products for the treatment or prevention of degenerative brain diseases.

Source	Active Compounds	Main Effect	Mechanism	Animal Model	Ref
*Allium cepa* (flesh and peel)	Quercetin-4′-glucoside Quercetin	Elevating cognitive behaviors (PAT, Y-maze, and MWM) Decreasing AChE activity Improving ROS-mediated oxidative stress (SOD, GSSG, GSH, and MDA levels)	AChE inhibition Antioxidant activity	Trimethyltin-induced cognitive deficits in ICR mice	[92]
*Arctium lappa* (root)	Arctigenin	Elevating cognitive behaviors (NORT and MWM) Decreasing Aβ activity via decreasing BACE1 and AEP levels Reducing pro-inflammatory cytokines	Aβ inhibition Anti-inflammatory activity TLR4/NF-κB signaling pathway	LPS-induced cognitive deficits in C57BL/6 mice	[96]
*Panax ginseng*	Ginsenoside	Elevating cognitive behaviors (PAT, Y-maze, and NORT) Decreasing AChE activity Inhibiting microglia activation	AChE inhibition	Aβ oligomer-induced cognitive deficits in ICR mice	[99]
*Stephania japonica* (stems)	Chloroform fraction of *S. japonica*	Elevating cognitive behaviors (MWM) Decreasing AChE activity Improving ROS-mediated oxidative stress (rGSH, MDA, SOD, and CAT levels)	AChE inhibition Antioxidant activity	Scopolamine-induced cognitive deficits in Swiss Albino mice	[102]
*Cucuma longa*	Curcuminoids	Elevating cognitive behaviors (PAT and MWM) Decreasing AChE activity Increasing BDNF expression Reducing NO, PGE2, iNOS, and COX-2 expressions	AChE inhibition BDNF-CREB-TrkB pathway Anti-inflammatory activity	Scopolamine-induced cognitive deficits in ICR mice	[107]
*Withania somnifera* (root)	Withanone	Elevating cognitive behaviors (PAT and EPM) Decreasing AChE activity Reducing pro-inflammatory cytokines (TNF-α, IL-1β, IL-6, and MCP-1) Reducing β- and γ-secretase enzymatic activity.	AChE inhibition Anti-inflammatory activity	STZ- induced cognitive deficits in Wistar rats	[114]
*Moringa oleifera* (leaf)	Methanol fraction of *M. oleifera*	Elevating cognitive behaviors (OFT, NORT, and WMZ) and synaptic plasticity Decreasing Aβ activity via decreasing BACE1 and AEP levels	Aβ inhibition Improving GluN2B via decreasing STEP activity Enhancing PSD95 and synapsin1 levels	AD mode of APP/PS1 mice	[119]
*Melissa officinalis*	Rosmarinic acid	Elevating cognitive behaviors (PAT and Y-maze) Increasing BDNF and NO synthase 3 genes expression	BDNF-CREB-TrkB pathway Enhancing NO synthase 3 gene	STZ-induced cognitive deficits in Wistar rats	[127]
*Salvia officinalis* (leaf)	Ethanol fraction of *S. officinalis*	Elevating cognitive behaviors (PAT) Improving ROS-mediated oxidative stress (MDA, SOD, and CAT levels)	Antioxidant activity	STZ-induced cognitive deficits in Wistar rats	[131]
*Rhodiola crenulata* (root)	salidroside	Elevating cognitive behaviors (Y-maze) Decreasing AChE activity Inhibiting Aβ and tau revels Improving ROS-mediated oxidative stress (MDA and SOD levels)	Aβ and tau inhibition Antioxidant activity Decreasing GSK3-β	Aβ oligomer-induced cognitive deficits in SD rats	[141]
*Cinnamomum verum*	Eugenol	Elevating cognitive behaviors (T-maze) Decreasing Aβ plaque in cerebellar histology	Aβ inhibition	AlCl_3_-induced cognitive deficits in Wistar rats	[145]
*Vitis vinifera* (stembark)	Vitisin A	Elevating cognitive behaviors (OFT, PAT, and Y-maze) Increasing BDNF, CREB, TrkB, and AKT expressions	BDNF-CREB-TrkB pathway	Scopolamine-induced cognitive deficits in C57BL/6 mice	[150]
*Magnolia officinalis*	Honokiol	Elevating cognitive behaviors (PAT and MWM) Decreasing Aβ activity via decreasing BACE1 and APP levels Reducing β-secretase enzymatic activity	Aβ inhibition	AD mode of Tg2576 mice	[152]
*Myristica fragrans*	Gallic acid	Elevating cognitive behavior (EPM, and PAT) Decreasing AChE activity Improving ROS-mediated oxidative stress (SOD, CAT, and GSH levels) Reducing TNFα, IL-β, and iNOS expressions Increasing HO-1 expression	AChE inhibition Antioxidant activity Anti-inflammatory activity Anti-apoptosis	Scopolamine-induced cognitive deficits in mice Scopolamine-induced cognitive deficits in Westar rats	[158,190]
*Vaccinium angustifolium*	Chlorogenic acid	Elevating cognitive behaviors (Y-maze, and PAT) Decreasing lipid peroxidation and MDA levels Decreasing AChE activity	AChE inhibition Antioxidant activity	Aβ oligomer-induced cognitive deficits in ICR mice	[191]
*Tinospora cordifolia*	Tinosporicide	Preventing anxiety, cognition, and motor coordination (NOR, EPM, and Rotarod test) Protecting hippocampal neurodegeneration and impaired synaptic plasticity (increasing MAP2, PSA-NCAM, Bcl-xL expression) Upregulating ERK and AKT pathways in the hippocampus Reducing pro-inflammatory cytokines (TNF-α, IL-1β, and IL-6)	Hippocampal neurogenesis Anti-inflammatory activity Modulation of NMDAR pathway, MEK/ERK and PI3K/AKT pathway	Glutamate-induced excitotoxicity in Wistar albino rats	[168]
*Arbutus unedo* (leaf)		Elevating cognitive behavior (MWM) Improving ROS-mediated oxidative stress (ROS, MDA, SOD, and CAT levels) Decreasing IL-6, TNF-α, caspase-3, and caspase-9 expression in the hippocampus Upregulating Nrf2, HO-1 expression	Hippocampal neurogenesis Antioxidant activity Anti-inflammatory activity Nrf2/HO-1 signaling pathway	STZ-induced cognitive deficits in rats	[192]
*Juglans regia*	Pedunculagin, casuarinin isomer	Elevating cognitive behaviors (Y-maze, PAT, and MWM) Improving ROS-mediated oxidative stress (MDA, SOD, and CAT levels) Restoring ACh, AChE, ChAT levels Increasing MMP levels and ATP contens of mitochondria in the brain Upregulating ZO-1 and Occludin expression Decreasing inflammatory protein expressions (TNF-α, TNFP1, p-JNK, p-IκB, COX-2, IL-1β, and caspase-3) Upregulating HO-1 and p-AKT	AChE inhibition Antioxidant activity Anti-mitochondrial dysfunction Protective effect of BBB dysfunction Anti-inflammatory activity AKT pathway	Aβ oligomer-induced cognitive deficits in ICR mice	[176]
*Acanthopanax senticosus*		Elevating cognitive behavior (NOR) Decreasing APP, NTRK1, ESR1, CFTR, CSNK2A1, EGFR, ESR2, GSK3B, and PAK1 expression Restoring TRAF6, p-MAP3K7, p-P38, and HSP27 levels	MAPK signaling pathway	5xFAD Tg mice	[183]
*Rosmarinus officinalis*	Eucalyptol (1,8-Cineole)	Elevating cognitive behavior (RAM, and PAT) Upregulating BDNF levels Increasing DCX-positive cells in DG	Hippocampal neurogenesis Enhancing BDNF	Scopolamine-induced cognitive deficits in Wister albino rats	[187]

## 5. Methods

We searched reference lists for studies published up to 10 September 2024, using the keywords ‘degenerative brain disease’, ‘Alzheimer’s disease’, ‘neuroprotection’, ‘reactive oxygen species’, and ‘natural products’. The search was conducted across multiple databases, including PubMed, Scopus, and the Web of Science. No restrictions were applied to sources or study designs, and we excluded articles published in languages other than English. Studies published between January 2000 and September 2024 were included.

## 6. Conclusions

Neurodegenerative diseases, such as AD, PD, HD, and ALS, are characterized by a complex multifactorial pathogenesis involving oxidative stress, mitochondrial dysfunction, and neuroinflammation, posing significant difficulties for understanding and therapeutic intervention. As the global prevalence of these diseases increases with the aging population, there is a growing need for novel and effective therapeutic interventions. Current pharmacological treatments are limited in their ability to modify disease progression, emphasizing the need for alternative approaches that target the underlying pathophysiological mechanisms. This review examines the critical role of ROS in the pathogenesis of neurodegenerative diseases, highlighting how excessive ROS production contributes to neuronal death through oxidative stress, excitotoxicity, and dysregulation of intracellular signaling pathways. Natural products have shown potential in suppressing ROS production, modulating inflammatory cascades, and promoting the activation of neuroprotective pathways such as the PI3K/AKT and BDNF-TrkB-CREB signaling axes. These pathways are essential for neuronal survival, synaptic plasticity, and the prevention of neurodegeneration. Moreover, natural products may influence key neurodegenerative processes by reducing Aβ and tau pathology, while also enhancing cholinergic neurotransmission through the inhibition of acetylcholinesterase activity. Further research is required to elucidate the precise molecular mechanisms by which these compounds exert their effects and to validate their efficacy in large-scale, randomized controlled trials. Integrating natural products into therapeutic regimens may offer a multifaceted approach to mitigating the progression and burden of neurodegenerative diseases, ultimately improving patient outcomes. Also, we believe that this review provides a foundational overview for investigating the efficacy of these treatments and suggests that natural products could be promising therapeutic candidates for the prevention of degenerative brain diseases.

## Data Availability

Not applicable.
